# Complex Dietary Topologies in Non-alcoholic Fatty Liver Disease: A Network Science Analysis

**DOI:** 10.3389/fnut.2020.579086

**Published:** 2020-09-29

**Authors:** Yang Xia, Zhiying Zhao, Shunming Zhang, Yashu Liu, Ge Meng, Qing Zhang, Li Liu, Hongmei Wu, Yeqing Gu, Yawen Wang, Tingjing Zhang, Xing Wang, Shaomei Sun, Ming Zhou, Qiyu Jia, Kun Song, Qijun Wu, Kaijun Niu, Yuhong Zhao

**Affiliations:** ^1^Department of Clinical Epidemiology, Shengjing Hospital of China Medical University, Shenyang, China; ^2^Nutritional Epidemiology Institute and School of Public Health, Tianjin Medical University, Tianjin, China; ^3^School of Computer Science and Engineering, Northeastern University, Shenyang, China; ^4^Department of Toxicology and Sanitary Chemistry, School of Public Health, Tianjin Medical University, Tianjin, China; ^5^Health Management Centre, Tianjin Medical University General Hospital, Tianjin, China; ^6^Tianjin Key Laboratory of Environment, Nutrition and Public Health, Tianjin, China; ^7^Center for International Collaborative Research on Environment, Nutrition and Public Health, Tianjin, China

**Keywords:** NAFLD, dietary network, network science, propensity score matching, case-control

## Abstract

**Background and Aims:** Previous studies have explored the associations between nutrition (food groups, nutrients, and dietary patterns) and the prevalence of non-alcoholic fatty liver disease. However, it remains unclear whether how foods are consumed together is associated with non-alcoholic fatty liver disease. The present study aims to construct dietary networks from network science and to explore the associations between complex dietary networks and non-alcoholic fatty liver disease.

**Methods:** The present case–control study generated 2,043 multivariate matched controls for 2,043 newly diagnosed non-alcoholic fatty liver disease cases. Mutual information, which represents both linear and non-linear dependencies among food groups, was used to construct the network topologies.

**Results:** The dietary topologies in the studied case and control groups were different despite the fact that only few food groups show differences in absolute intake. The dietary structure of the case group focused on two major components with more cohesion among food groups, while contrarily the control group had one major component with higher diversity of food groups. The dietary topology of the case group showed equality in connections among beneficial and detrimental food groups, whereas the control group focused more on healthier food choices.

**Conclusions:** This study suggests how foods are consumed, besides the absolute intake, could be an important determinant of the occurrence of non-alcoholic fatty liver disease. A diverse diet that focuses on whole grain, tubers, and vegetables could yield beneficial effects regarding non-alcoholic fatty liver disease. Network science could offer a complementary tool in nutritional epidemiology.

## Keypoints

- How foods are consumed, besides the absolute intake, could be an important determinant of the occurrence of non-alcoholic fatty liver disease.
- Network science could offer a complementary tool in nutritional epidemiology.

## Introduction

Non-alcoholic fatty liver disease (NAFLD) develops without alcohol abuse. It is defined as the presence of at least 5% hepatic steatosis without evidence of hepatocellular injury in the form of hepatocyte ballooning (1). As reported in a meta-analysis conducted in 2016, 25% of the global adult population were afflicted with NAFLD (2). NAFLD constitutes not only a potentially progressive course that leads to liver fibrosis, cirrhosis, hepatocellular carcinoma, and liver transplantation (3) but is also associated with other non-communicable diseases, such as type 2 diabetes (4) and cardiovascular diseases (5). Considering the increasing disease burden of NAFLD, it is important to identify risk factors and develop appropriate treatment strategies. Lifestyle interventions, particularly a healthy diet, have been recognized as effective treatments in early to advanced stages of NAFLD (1).

Previous studies have explored the associations between NAFLD and the intake of nutrients and food items, such as mushrooms (6), yogurts (7), raw garlic (8), nuts (9), oranges (10), soft drinks (11), dietary fibers (12), and fructose (13). Moreover, dietary patterns, which encompass the effects of overall diet and closely parallels the real world situation (14), were also shown to be associated with the prevalence of NAFLD (15–22). In fact, our previous study has shown that higher carbohydrate/sweet pattern scores are associated with a higher prevalence of NAFLD among females (16). Another study has also demonstrated that higher intake of a healthy dietary pattern (characterized by higher intake of fruits, vegetables/legumes, white meats, olive oil, margarine, and bread/toast) is associated with a lower prevalence of NAFLD (20). It should be noted, however, that all the aforementioned dietary patterns assessed in previous studies were derived based on the hypothesis that the associations among the intakes of food items were linear. For example, factor analysis reduces data into patterns that can explain the maximum variation in food intake based on linear inter-correlations between dietary items (23). But reduced rank regression focuses on identifying linear functions of food groups, which explains as much variation as possible in a set of intermediate response variables (24). However, the associations between food item intakes could be non-linear. A recent study from the network science approach has derived some dietary patterns that fully reflect the complex interconnectedness of food intakes, and explored the associations between dietary patterns and dementia (25). Networks are data-based mathematical models of complex systems that can identify both linear and non-linear associations and explore complex dynamics (26). Compared with traditional statistical methods used in the derivation of dietary patterns, network science can help discover the potential role of food groups in overall dietary patterns and provide a new insight into the complexity, particularly the non-linearity, of dietary patterns (25). To the best of our knowledge, no study has been conducted to explore the association between NAFLD and dietary patterns constructed based on non-linear associations among food groups. Moreover, no study has been carried out to investigate the differences in comprehensive interactions among food groups between NAFLD patients and their controls, referred to as the case group and the control group, respectively. These are the topics and also the main contributions of the present study, which aims at a case-control study to explore the differences in dietary pattern structures between NAFLD patients and their controls using network science tools.

## Methods

### Participants

The present case–control study was conducted based on the Tianjin Chronic Low-grade Systemic Inflammation and Health (TCLSIHealth) Cohort Study, a large prospective dynamic cohort study focusing on the associations between chronic low-grade systemic inflammation and the healthy status of a population living in Tianjin, China (15, 16). Participants were recruited when they were taking annual health examinations at the Tianjin Medical University General Hospital-Health Management Center and some other community management centers in Tianjin.

**Figure 1:**
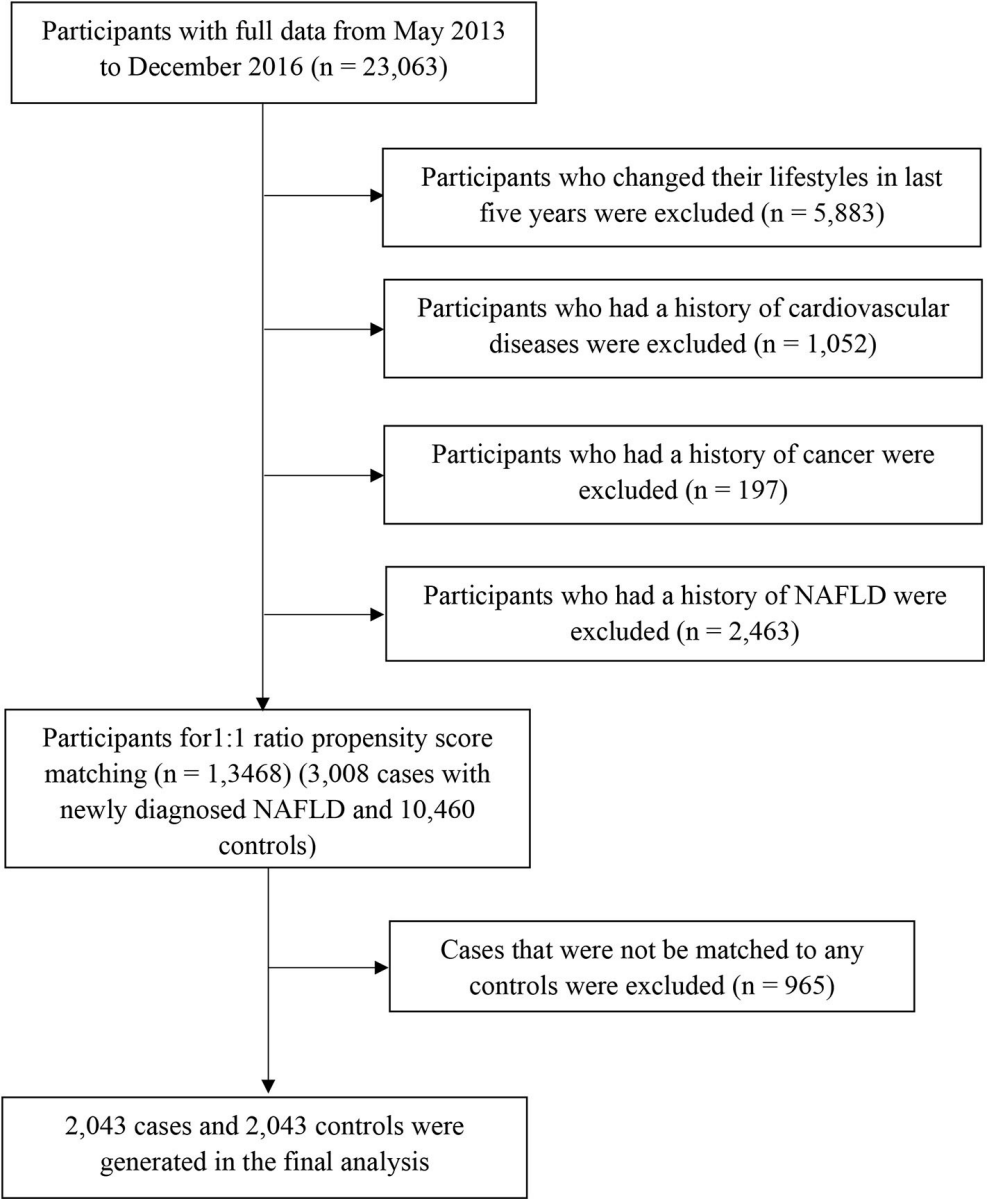
Flow diagram of study participant selection. NAFLD, non-alcoholic fatty liver disease.

Participants with missing variables or those with implausible energy intakes (≤400 or ≥10,000 kcal/day) were excluded (*n* = 1,521) in the process of data clean. Afterall, 23,063 participants without acute inflammatory disease completed comprehensive health examinations and answered questionnaires between May 2013 and December 2016. We excluded participants who changed their lifestyles (e.g., diet, drinking, smoking, physical activity, and sleeping) in the last 5 years (*n* = 5,883) or those with a history of cardiovascular diseases (*n* = 1,052) or cancer (*n* = 197). We also excluded participants who had a history of NAFLD (*n* = 2,463). As a result, the final population comprised 13,468 participants (3,008 cases with newly diagnosed NAFLD and 10,460 controls) for propensity score matching (Figure 1). The study protocol was approved by the Institutional Review Board of the Tianjin Medical University. All participants provided written informed consent prior to enrolment in the study.

### Propensity Score Matching

Propensity scores were calculated for all participants using a logistic regression model with the following covariates: sex, age, body mass index (BMI), physical activity, energy intake, education level, household income, smoking status, drinking status, employment status, metabolic syndrome status, and family history of cardiovascular disease, hypertension, and diabetes. Using these propensity scores, cases were individually matched to control using the nearest matching method within a caliper distance, which selects for matching a control subject whose propensity score is closest to that of the case subject. This is known as the nearest neighbor matching approach. Moreover, a further restriction is imposed, where the absolute differences in propensity scores of matched subjects must be below some pre-specified threshold (the caliper distance) (27). Thus, participants, for whom the propensity score could not be matched due to a greater caliper distance, were excluded from further analysis. As suggested by Austin (27), a caliper width equal to 0.2 of the standard deviation of the logit of the propensity score was used, because this value minimizes the mean squared error of the estimated treatment effects in several scenarios. To better match cases and controls, we used the 1:1 ratio matching method. Cases that could not be matched to any controls were discarded. Finally, 2,043 cases and 2,043 controls were generated using this propensity score matching method (Figure 1).

### Assessment of Dietary Intake

Dietary intake was assessed using a modified version of the food frequency questionnaire (FFQ) that includes 100 food items [the initial version of the FFQ included only 81 food items (16)] with specified serving sizes. The FFQ includes seven frequency categories ranging from “almost never eat” to “twice or more per day” for foods and eight frequency categories ranging from “almost never drink” to “four or more times per day” for beverages. The reproducibility and validity of the questionnaire were assessed with a random sample of 150 participants from our cohort using data from repeated measurements of the FFQ ~3 months apart, and 4-d weighed diet records (WDR). The Spearman rank correlation coefficient for energy intake between the two FFQs was 0.68 (*P* < 0.05). The correlation coefficients for food items (i.e., fruits, vegetables, fish, meat, and beverages) between the two FFQs were ranged from 0.62 to 0.79 (all *P* < 0.05). Meanwhile, the Spearman rank correlation coefficient for energy intake assessed using the WDR and FFQ was 0.49 (*P* < 0.05). Correlation coefficients for nutrients (i.e., vitamin C, vitamin E, polyunsaturated fatty acid, saturated fatty acids, carbohydrates, and calcium) were assessed using the WDR and FFQ ranged from 0.35 to 0.54 and from 0.39 to 0.72 before and after adjustments for energy intake, respectively (all *P* < 0.05). The mean daily intakes of nutrients were calculated using an *ad-hoc* computer program developed to analyse the questionnaire responses. Consumption of food items was calculated by multiplying the portion size (g/time) by the frequency with which each food item was consumed per day. Furthermore, Chinese food composition tables (28) were used as the nutrient database to calculate nutrient intakes. Nutrient intake was calculated by first multiplying the amount (in grams) consumed for each food item with its nutrient content per gram and then adding the nutrient contributions across all food items. Similar food items were further collapsed into 25 food groups based on the characteristics of food items for network science analyses.

### Liver Ultrasonography and Definitions of NAFLD

Liver ultrasonography was performed by trained sonographers using a TOSHIBA SSA-660A ultrasound machine (Toshiba, Tokyo, Japan), with a 2–5 MHz curved array probe. According to the revised definition and treatment guidelines for NAFLD put forth by the Chinese Association for the Study of Liver Disease in 2010 (29), we define “heavy drinking” as >140 g alcohol intake per week in men and >70 g per week in women. Total alcohol intake in the past week was assessed using the FFQ. Participants were diagnosed as having NAFLD using abdominal ultrasonography (evidenced by brightness of the liver and a diffusely echogenic change in the liver parenchyma) and no history of heavy drinking. Participants with a history of self-reported or previously diagnosed NAFLD were excluded in the present study. Thus, all participants with NAFLD in the present study were newly diagnosed cases.

### Assessment and Definition of Matching Variables

Sociodemographic variables (including sex, age, education, employment status, smoking status, drinking status, and household income) were also assessed using the questionnaire. The educational level was assessed by asking the question “what is the highest degree you earned?,” which was divided into two categories: < college graduate or ≥college graduate. Employment statuses were classified as either senior officials and managers or professionals. Information on smoking status (“never,” “former,” and “current smoking”) and drinking status (“never,” “former,” “current drinking everyday,” and “current drinking sometime”) among the participants was obtained from the questionnaire survey. Physical activity in the most recent week was assessed using the short form of the International Physical Activity Questionnaire (IPAQ) (30). BMI (in kg/m^2^) was calculated by dividing the weight (in kilograms) by the square of the height (in meters). Waist circumference was measured at the umbilical level with participants standing and breathing normally. The blood pressure was measured twice in the left upper arm using a TM-2655P automatic monitor (A&D Co., Tokyo, Japan) in a seated position, with a 5-min rest in between. The mean of these two measurements was taken as the blood pressure value.

Fasting blood samples were obtained via venepuncture of the cubital vein and immediately mixed with ethylenediaminetetraacetic acid. Fasting blood glucose concentrations were measured using the glucose oxidase method, triglyceride levels were measured using enzymatic methods, and high-density lipoprotein cholesterol levels were measured using the chemical precipitation method with reagents from Roche Diagnostics GmbH (Mannheim, Germany) on an automatic biochemistry analyser (Roche Cobas 8000 modular analyzer). Finally, metabolic syndrome was defined in accordance with the criteria of the American Heart Association scientific statement of 2009 (31).

### Statistical Analysis

The networks of dietary patterns among NAFLD patients and controls were built using mutual information (MI), which was used to infer the associations among food groups. MI measures the information shared by two discrete random variables. It measures how much knowing one of these variables reduces the uncertainty about the other (32). It quantifies the amount of information obtained about one random variable *X* through the other random variable *Y* by determining how similar the joint distribution *p*(*x, y*) is to the products of the factored marginal distributions, *p*(*x*)*p*(*y*) (25):

$$\begin{array}{l} MI\left(X;Y\right)=\underset{x,y}{\sum }p\left(x,y\right)\log\frac{p\left(x,y\right)}{p\left(x\right)p\left(y\right)} \end{array}$$

The MI is non-negative and symmetric in *X* and *Y*. The MI is zero when *X* is independent of *Y*. Compared with traditional correlation measures, which capture only linear dependence, the MI contains information about both linear and non-linear dependencies (33).

First, we computed the MI matrix for cases and controls using the Miller–Madow estimator (34) using the *build.mim* function in the *minet* R package (35). As suggested by Meyer (35), considering that the intakes of food groups were continuous variables, we partitioned the intake of food groups into subintervals with equal frequencies, called bins. The number of bins to be used for discretisation is set by default to $\sqrt{m}$ where *m* is the number of samples (36). The MI matrices for the case and control groups are presented in Supplementary Figures 1, 2, respectively.

Second, the edge score for each pair of food groups in each network was inferred using the *mrnet* inference algorithm (37) in the *minet* R package. This function takes the MI matrix as an input and returns the adjusted MI values in the form of a weighted adjacency matrix of the network. Weights >0 can be interpreted as implying higher confidence associations (25).

For visualization, the landscapes of food intake networks for the case and control groups were contributed by food groups as nodes and the associations between them as edges. Furthermore, since the adjusted MI values were displayed in the form of a weighted adjacency matrix of the network, the weights of edges were set using values obtained directly from the matrix. The width of the edge was set proportional to the weight of connections (for better interpretability, plots were limited to edges with inferred weight ≥0.30), and the node size was set proportional to the absolute intake of each corresponding food group. The colors from light to dark were proportional to the strengths of the nodes.

The structural properties of the networks were calculated by both weighted degrees (namely, the strength) and hubs (similar to authorities in undirected networks). The strength was calculated by summing up the edge weights of the adjacent edges for each node (38). The hub scores of the nodes were defined as the principal eigenvector **A** × *t*(**A**), where **A** is the adjacency matrix of the graph (39). Compared with strength, which represents the direct association between each node and the others, a hub can describe the importance of a node considering both itself and all the nodes to which it is connected, computed via an iterative algorithm that maintains and updates numerical weights for each node. In conclusion, strength represents direct the interaction of each node with the others, while hub can be used to measure the importance of each node in the entire network. The differences in strength and hub for each food group between the case and control groups were calculated (by subtracting the values in the control group from those in the case group). All the above statistical analyses were performed using SAS version 9.4 for Windows (SAS Institute Inc., Cary, NC, USA) and the *minet* package in the R environment (version 4.0; R Development Core Team, Vienna, Austria). The topologies of networks were visualized using Gephi version 0.9.2 for Windows (www.gephi.org).

## Results

### Characteristics of Participants

The characteristics of participants before matching are presented in Supplementary Table 1. Among the 13,468 participants, 22.3% were classified as having newly diagnosed NAFLD. Participants with NAFLD were mostly men, older, current smokers, ex-smokers, and current drinkers, many also with metabolic syndrome, higher levels of BMIs, daily energy intakes, alanine aminotransferase, aspartate aminotransferase, and γ-glutamyl transpeptidase, lower education levels, unlikely managers, and had a family history of diabetes (all *P* < 0.05). The characteristics of participants (2043 NAFLD cases and 2043 controls) after matching are presented in Table 1. There were no significant differences in matching variables between the case and the control groups.

**Table 1:** Participant characteristics by NAFLD status after matching^a^.

**Characteristics**	**NAFLD status**		***P*-value^**b**^**
	**No (*n* = 2,043)**	**Yes (*n* = 2,043)**	
Sex (male%)	68.6	69.6	0.48
Age (y)	45.1 (44.5, 45.6)^c^	44.6 (44.1, 45.1)	0.22
BMI	26.5 (26.4, 26.6)	26.6 (26.5, 26.7)	0.19
Metabolic syndromes (%)	42.4	44.0	0.31
Physical activity (Mets × hours/week)	10.2 (9.6, 10.8)	10.1 (9.5, 10.7)	0.83
Energy intake (kJ/d)	8247.0 (8149.0, 8346.6)	8252.4 (8154.0, 8352.0)	0.94
Education (≥college graduate, %)	51.6	53.5	0.22
Household income (≥10,000 Yuan, %)	33.8	34.7	0.54
ALT (U/L)	19.6 (18.7, 20.4)	31.9 (31.0, 32.8)	<0.0001
AST (U/L)	18.5 (17.9, 19.0)	22.0 (21.5, 22.6)	<0.0001
GGT (U/L)	30.9 (26.4, 35.4)	45.4 (40.8, 49.9)	<0.0001
**Smoking status (%)**			
Smoker	29.0	29.1	0.94
Ex-smoker	7.6	7.6	1.00
Non-smoker	63.4	63.3	0.95
**Drinker (%)**			
Everyday	8.4	9.1	0.46
Sometime	58.8	59.9	0.49
Ex-drinker	7.9	8.1	0.76
Non-drinker	24.9	22.9	0.14
**Employment status (%)**			
Managers	39.7	40.2	0.79
Professionals	17.6	17.0	0.61
Other	42.7	42.9	0.90
**Family history of diseases (%)**			
CVD	28.3	27.8	0.68
Hypertension	50.4	49.9	0.73
Diabetes	23.4	23.5	0.91

a *NAFLD, non-alcoholic fatty liver disease; CVD, cardiovascular disease; BMI, body mass index; ALT, alanine aminotransferase; AST, aspartate aminotransferase; GGT, γ-glutamyl transpeptidase*.

b *Analysis of variance or chi-square test*.

c *Least square mean (95% confidence interval) (all such values)*.

The average intake of food groups according to NAFLD status are presented in Table 2. Participants with NAFLD showed higher intakes of fish, ice cream and candy, tea and tea beverages, and sugar-containing beverages but lower intakes of whole grain (all *P* < 0.05).

**Table 2:** Average intake of food groups according to NAFLD status^a^.

**Food groups**	**NAFLD status**		***P*-value^**b**^**
	**No (*n* = 2,043)**	**Yes (*n* = 2,043)**	
Refined grain	352.98 (344.83, 361.12)^c^	356.71 (348.57, 364.86)	0.52
Whole grain	218.64 (211.48, 225.81)	197.27 (190.10, 204.44)	<0.0001
Dairy	136.97 (130.13, 143.80)	127.49 (120.65, 134.32)	0.05
Meat	65.61 (63.23, 67.99)	68.36 (65.98, 70.74)	0.11
Animal organs	14.99 (13.75, 16.23)	15.31 (14.07, 16.56)	0.72
Fish	41.19 (38.95, 43.43)	45.01 (42.77, 47.24)	0.02
Egg	37.78 (36.80, 38.75)	37.60 (36.62, 38.57)	0.80
Preserved egg	2.98 (2.61, 3.36)	3.06 (2.69, 3.44)	0.77
Fruits	382.82 (365.07, 400.58)	406.05 (388.30, 423.80)	0.07
Vegetables	250.62 (243.53, 257.71)	248.12 (241.03, 255.21)	0.62
Tubers	48.61 (46.20, 51.01)	48.20 (45.80, 50.61)	0.82
Legume and legume products	44.64 (42.92, 46.35)	43.60 (41.88, 45.32)	0.40
Pickled foods	11.12 (10.28, 11.97)	12.08 (11.23, 12.93)	0.12
Western-style cake, cookie	14.79 (13.48, 16.10)	16.36 (15.05, 17.67)	0.10
Ginger and garlic	5.78 (5.52, 6.05)	5.63 (5.37, 5.90)	0.44
Ice cream and candy	11.44 (10.00, 12.88)	13.84 (12.40, 15.29)	0.02
Nuts	9.04 (8.50, 9.59)	9.17 (8.63, 9.71)	0.75
Tea and tea beverages	229.64 (213.80, 245.48)	257.23 (241.39, 273.07)	0.02
Coffee	26.27 (22.94, 29.59)	26.48 (23.15, 29.81)	0.93
Sugar-containing beverages	22.90 (19.68, 26.13)	29.76 (26.53, 32.98)	<0.001
Fruits and vegetables juice	23.38 (20.74, 26.02)	26.67 (24.02, 29.31)	0.08
Alcohol and alcoholic beverages	79.15 (71.41, 86.89)	81.51 (73.78, 89.25)	0.67
Processed meat	4.88 (4.54, 5.22)	4.93 (4.59, 5.27)	0.83
Chinese cake	11.08 (9.77, 12.39)	10.98 (9.68, 12.29)	0.92
Animal blood	3.02 (2.56, 3.47)	2.95 (2.49, 3.40)	0.83

a *NAFLD, non-alcoholic fatty liver disease*.

b *Analysis of variance*.

c *Least square mean (95% confidence interval) (all such values)*.

### Network Topologies of Dietary Patterns

**Figure 2:**
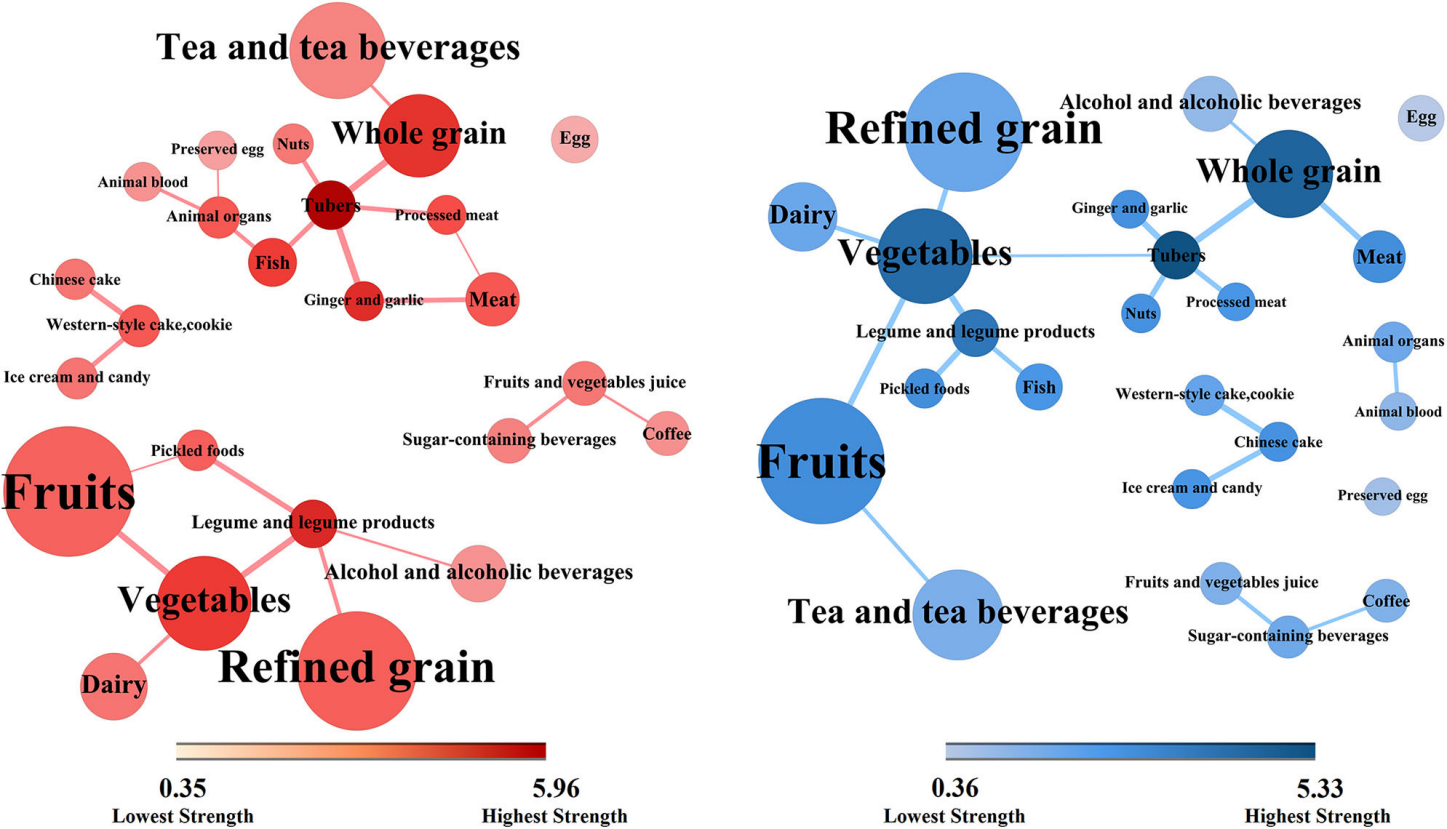
Dietary topologies among cases of non-alcoholic fatty liver disease (red) and matched controls (blue). Dietary topologies computed separately among cases of non-alcoholic fatty liver disease (red) and matched controls (blue) using mutual information. Edge width sets proportional to the weights of connections (for better interpretability, plots are limited to edges with inferred weight ≥0.30), and node size sets proportional to the absolute intake of each corresponding food group, where the colors from light to dark were proportional to the strengths of each nodes.

The network topologies of dietary patterns among cases and controls are presented in Figure 2 (case group, red; control group, blue). These network topologies showed the connections between food groups and the entire structure of the dietary patterns among case and control groups.

There were five components in the case group and six in the control group. However, there were two major components in the case group. The core nodes, which had high strengths and may play central roles in the first component were tubers, whole grain, ginger and garlic, fish, animal organs, meat, and processed meat. The core nodes in the second component comprised vegetables, legume and legume products, refined grain, and fruits. In contrast, there was only one major component in the control group. The core nodes comprised tubers, whole grain, vegetables, and fruits. Moreover, there were two small clusters of the food groups in both case and control groups. The first contained Chinese cakes, western-style cakes and cookies, and ice cream and candy. The second contained fruit and vegetable juices, sugar-containing beverages, and coffee.

Furthermore, there were more circles in the case topology than in the control topology. For example, tubers, processed meat, meat, and ginger and garlic, altogether form closed circle in the case group. The circles in the case topology suggested that the dietary habit presented more cohesively regarding connectivity while the control structure showed equality among most food groups in terms of topology.

### Differences in Networks Between Cases and Controls

We calculated the strengths and hubs for all food groups according to NAFLD status (Supplementary Table 2). The mean values of strengths in the case and the control groups were 2.32 and 2.40, respectively. The mean values of hubs in the case and the control groups were 0.37 and 0.41, respectively. Tubers yielded the highest strengths and hubs while eggs yielded the lowest values among both cases and controls.

**Figure 3:**
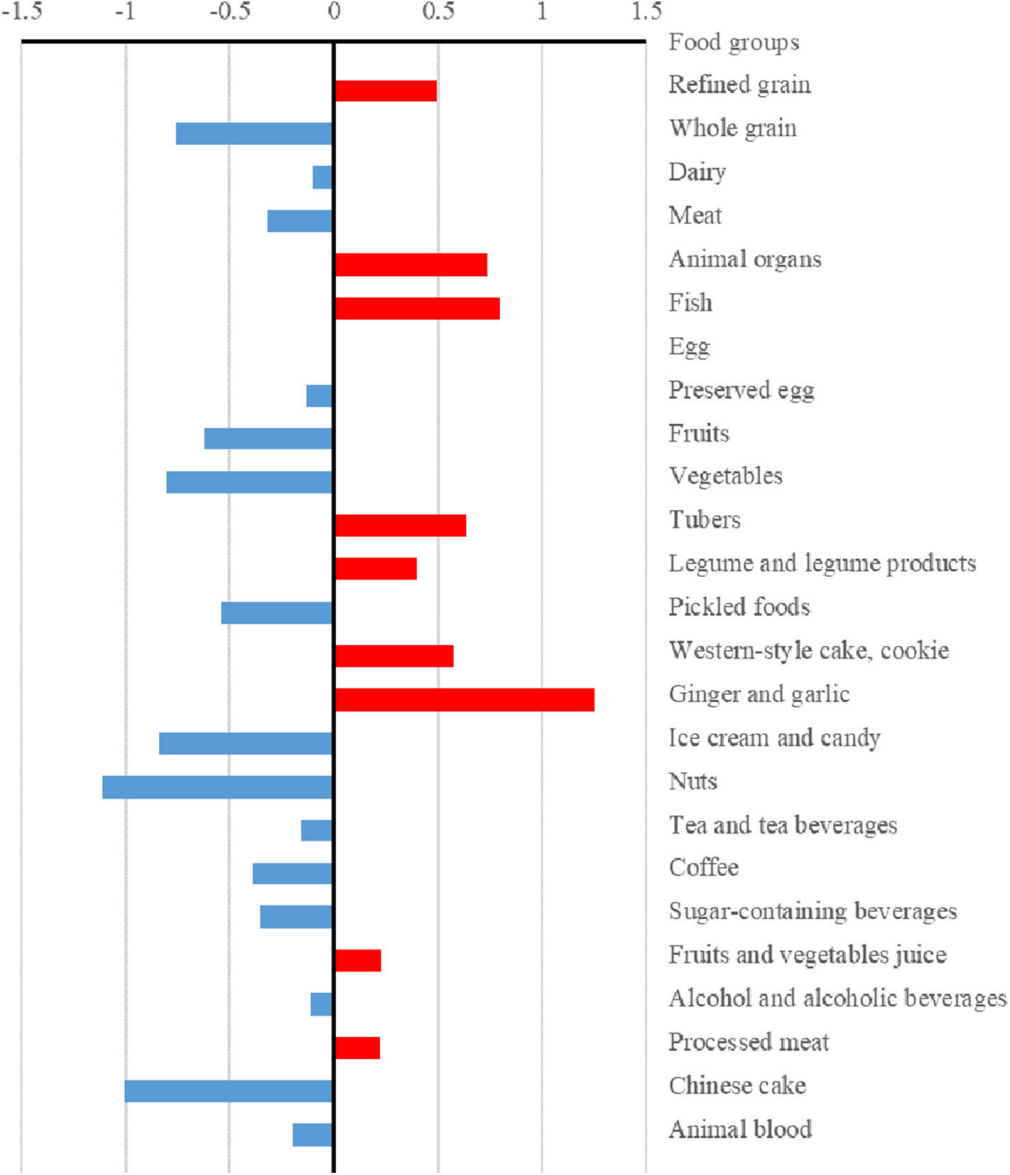
Differences in the strengths of food groups between case and control dietary topologies. For each node, strength was computed as the sum of edge weights (mutual information) associated with other nodes, which represented the direct associations between each node and others. The differences in strengths of food nodes calculated by subtracting control values from case values.

**Figure 4:**
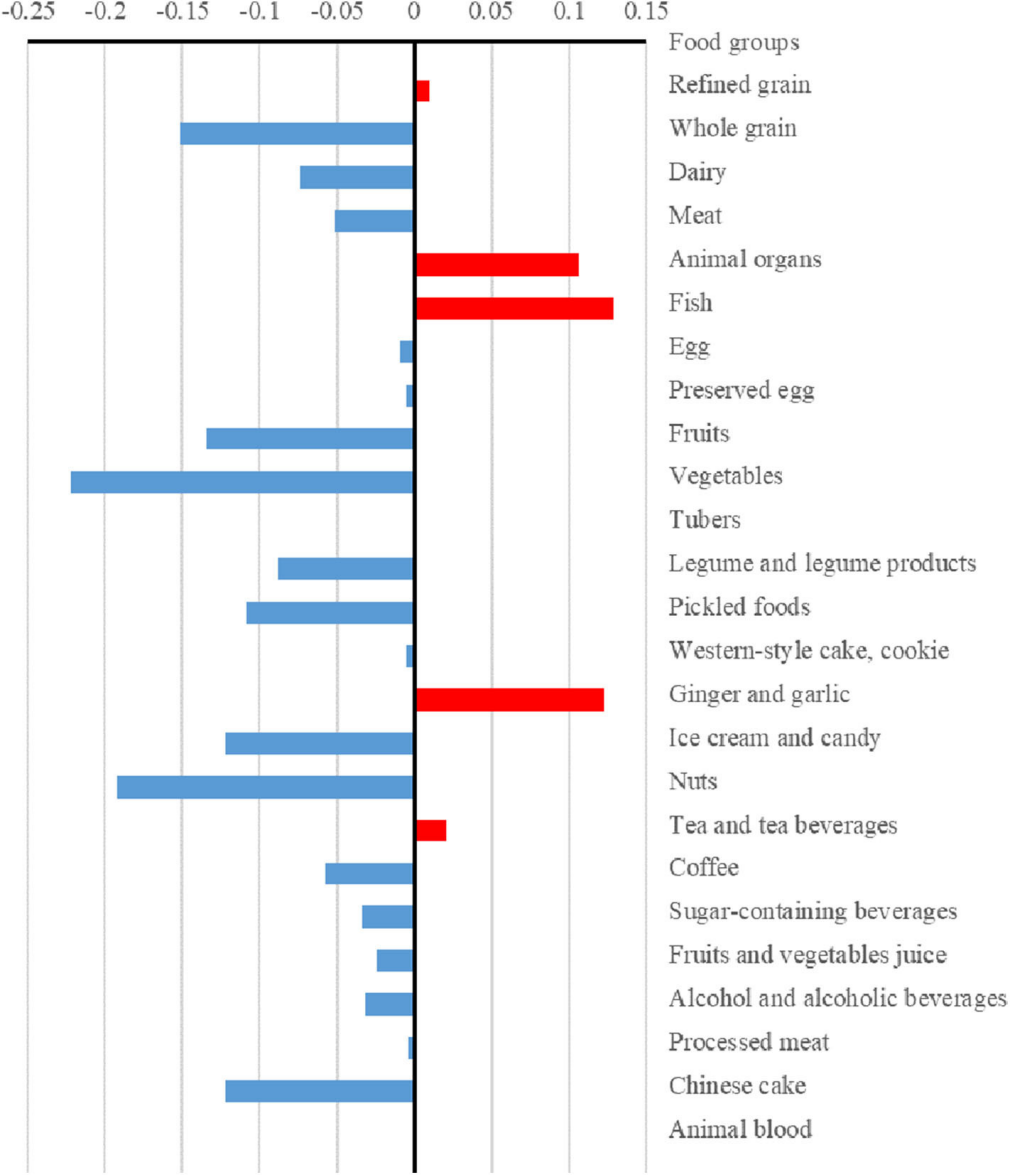
Differences in the hubs of food groups between case and control dietary topologies. For each node, hub was defined as the principal eigenvector of **A**×*t*(**A**), where **A** is the adjacency matrix of the graph. The hub can describe the importance of a node considering both itself and all connected nodes, which was computed via an iterative algorithm that maintains and updates numerical weights for each node. The differences in hubs for food nodes calculated by subtracting control values from case values.

The differences (case values minus control values) of the strengths and hubs in the food groups between cases and controls are presented in Figures 3, 4, respectively. Overall, the control group had higher strengths and, particularly, hubs in most food groups. For strengths, which represented the direct interactions of each food group with others, ginger and garlic yielded the largest positive value while nuts yielded the largest negative value. For hubs, which represented the importance of each food group in the entire network, fish yielded the largest positive value while vegetables yielded the largest negative value.

## Discussion

This study first used network science tools to explore the differences in dietary topologies between patients with NAFLD and controls. The dietary network topologies were constructed using MIs, which contain information about both linear and non-linear dependencies, among food groups (33). Further, the dietary network topologies provided information not only on simple associations among food groups but also on comprehensive interactions in dietary intake habits among participants. The results suggest that the dietary structures are different between the case and the control groups. The dietary structure of the case group focuses on two major components, whereas the control group has only one major component. In the case group, it was found that the dietary habits present more cohesively on connectivity in each component, whereas the control structure shows equality among most food groups. Besides absolute intake, food groups plays a role in the entire dietary structure and they are subsequently associated with NAFLD.

Previous studies have explored the associations between absolute intake of single food groups or nutrients and NAFLD. For example, a previous study found that consumption of raw garlic was inversely associated with NAFLD among Chinese men (8). Another study suggested that a higher intake of insoluble dietary fiber is associated with a lower prevalence of newly diagnosed NAFLD (12). In recent years, some studies have focused on the entire effect of diet and explored the associations between dietary patterns and NAFLD (15–22). For example, our previous study showed that animal food patterns was positively associated with the prevalence of NAFLD (15). However, in the above studies, the dietary pattern scores were calculated based on the absolute intake of food groups and their importance in dietary patterns. Thus, the dietary pattern was associated with NAFLD, implies in truth that the sum of weighted absolute intakes of food groups was associated with NAFLD. However, no study has explored the associations between how we eat foods as a whole (as opposed to how much we eat) and the prevalence of NAFLD. Only one previous study applied network science to explore the associations between complex dietary behaviors and dementia (25). The results suggested that how foods are consumed (but not only the quantity consumed) may be important for dementia prevention (25). In line with the previous study (25), we found that compared with studies that focused on single food groups, nutrients, or dietary patterns derived using traditional methods, network science provided an additional layer of complexity in the associations between dietary intake and NAFLD. For example, the results suggest that tubers were core nodes in network topologies among both cases and controls and there was no statistical difference in tuber intake between the two groups. However, in the case group, tuber intake was directly associated with several nodes with nearly equal hubs (whole grain, fish, processed meat, ginger, and garlic). But, in the control group, only two core nodes (whole grain and vegetables) were directly associated with tuber intake. This suggested that how tubers are consumed (not only the absolute intake) could be an important determinant of the NAFLD occurrence.

Moreover, at the level of the entire network, the results suggest that the dietary structure of the case group had two major components. Interestingly, the two major components in the case group were both characterized by food groups with beneficial and detrimental effects on NAFLD, while the hub nodes were equal. The core nodes in the first component comprised tubes, whole grain, ginger and garlic, fish, animal organs, meat, and processed meat. For example, a previous study showed that consumption of whole grains had beneficial effects on hepatic steatosis and liver enzymes concentrations among patients with NAFLD (40). Meanwhile, frequent consumption of raw garlic was also inversely associated with NAFLD among Chinese men (8). However, consumption of animal organs and meat was positively associated with NAFLD (15). Thus, although the absolute intakes of whole grain, garlic, animal organs, and meat were the same in the case and the control groups in the present study, the beneficial effects of whole grain and garlic could be covered by the detrimental effects of animal organs and meat in the case group, and vice versa. A similar structure was found in the second component in the case group, which was characterized by core nodes comprising legume and legume products, fruits, vegetables, and refined grain. The beneficial effects of legume and legume products, fruits, and vegetables could be covered by the intake of refined grain, and vice versa. However, in the control group, we found only one major component, typified by food groups with beneficial effects on NAFLD as hub nodes, such as whole grain, tubers, vegetables, and fruits.

Furthermore, we found that, compared with those of the case group, the control group had higher strengths and, particularly, hubs for most food groups. Meanwhile, there were more circles in the case topology (instead of stars in the control topology). We observed that whole grain, tubers, and vegetables were the core nodes as stars in the dietary structure in the control group. The results suggested that the dietary habits of the case group were focused on some specific food groups and circles of food groups while the control group showed a higher healthy diversity in food choices. Thus, a well-diversified diet that focuses on whole grain, tubers, and vegetables could yield beneficial effects regarding NAFLD. There are several plausible mechanisms underlying the results. First, a previous study suggested that higher healthy food diversity was inversely associated with the indicators of body adiposity in the United States (41). Meanwhile, high visceral adiposity was associated with high risk of NAFLD (42). Second, the hub food groups (whole grain, tubers, and vegetables) in the control group contain greater fiber, which leads to a slower digestion of macronutrients and have beneficial effect on blood glucose burden and insulin concentrations (43). Disruption of glucose and insulin play important role in the development of NAFLD (44, 45). Third, other components, such as polyphenols, in vegetables also contributed to the lower prevalence of NAFLD (46).

The use of network science to derive dietary patterns was the main strength of the present study. Compared to the methods previously used in the derivation of dietary patterns, the network topologies here are constructed using MI, which contains information about both linear and non-linear dependencies among food groups. Moreover, by comparing the network topologies between the case and the control groups, one could conclude, as dietary suggestions for preventing NAFLD based on an overall food system, that how to eat but not only how much to eat is very important. The second strength of the present study lies in the inclusion of participants who were newly diagnosed with NAFLD, and our exclusion of participants who had changed their lifestyles in the last 5 years. Based on these inclusion and exclusion criteria, the reverse causation (i.e., participants with NAFLD changing their diet to reduce weights) was corrected accordingly.

Nevertheless, this study had some limitations. First, recall bias may have arisen from our use of a self-reporting questionnaire. Second, using the network method, we were unable to explore the differences in network topologies between the case and the control groups at an individual level. Moreover, confounding factors could not be adjusted. For this reason, we used the propensity score matching method to balance the case and the control groups. Thus, as shown in Table 1, all measured matching factors were balanced between the two groups. Third, we used hepatic ultrasonography instead of liver biopsies to detect fatty liver, as liver biopsies were unavailable during health examinations of the target population in our data collection. A previous study has found that ultrasonography has a sensitivity of 89% and a specificity of 93% for detecting NAFLD, and is widely used in population-based studies due to its non-invasiveness and accessibility (47). Yet, ultrasonography has limited sensitivity and does not reliably detect steatosis when the amounts of fat are low or when individuals have high BMI.

## Conclusions

This study suggests that how foods are consumed, but not only the absolute intake, could be important in determining the occurrence of NAFLD. A diverse diet that focuses on whole grain, tubers, and vegetables could yield beneficial effects regarding NAFLD. Thus, despite absolute intake of food groups, dietary intervention strategies for NAFLD should also focused on whole dietary structures. Future randomized controlled trials that explore the effect of such dietary structures on NAFLD are needed to clarify the results in the present study. Moreover, it was demonstrated that network science could provide a complementary tool for in-depth studying nutritional epidemiology.

## Data Availability Statement

The raw data supporting the conclusions of this article will be made available by the authors, without undue reservation.

## Ethics Statement

The protocol of this study was approved by the Institutional Review Board of the Tianjin Medical University and participants gave written informed consent before participation in the study.

## Author Contributions

YZ, KN, and YX contributed to the study conception and design. YX, SZ, YL, GM, QZ, LL, HW, YG, YW, TZ, XW, SS, MZ, QJ, KS, and KN contributed to check the data and results. YX, ZZ, and QW contributed to the drafting and revising of the manuscript. KN and YZ contributed to the approval of the final version of the manuscript. All authors contributed to the article and approved the submitted version.

## Conflict of Interest

The authors declare that the research was conducted in the absence of any commercial or financial relationships that could be construed as a potential conflict of interest.

## Supplementary Material

Supplementary Figure 1 Mutual information matrix of cases. Light to dark colors indicate lower to higher values.

Supplementary Figure 2 Mutual information matrix of controls. Light to dark colors indicate lower to higher values.

Supplementary Table 1 Participant characteristics by NAFLD status before matching^a^.

Supplementary Table 2 Strengths and hubs for food groups according to NAFLD status^a^.

## Abbreviations

BMI: Body mass index
FFQ: Food frequency questionnaire
MI: Mutual information
NAFLD: Non-alcoholic fatty liver disease
WDR: Weighed diet records.
