# Supplementary figures and images for: Complex Dietary Topologies in Non-alcoholic Fatty Liver Disease: A Network Science Analysis
Source: Front Nutr. 2020 Sep 29;7:579086. doi: 10.3389/fnut.2020.579086 (PMC7557363; doi:10.3389/fnut.2020.579086)

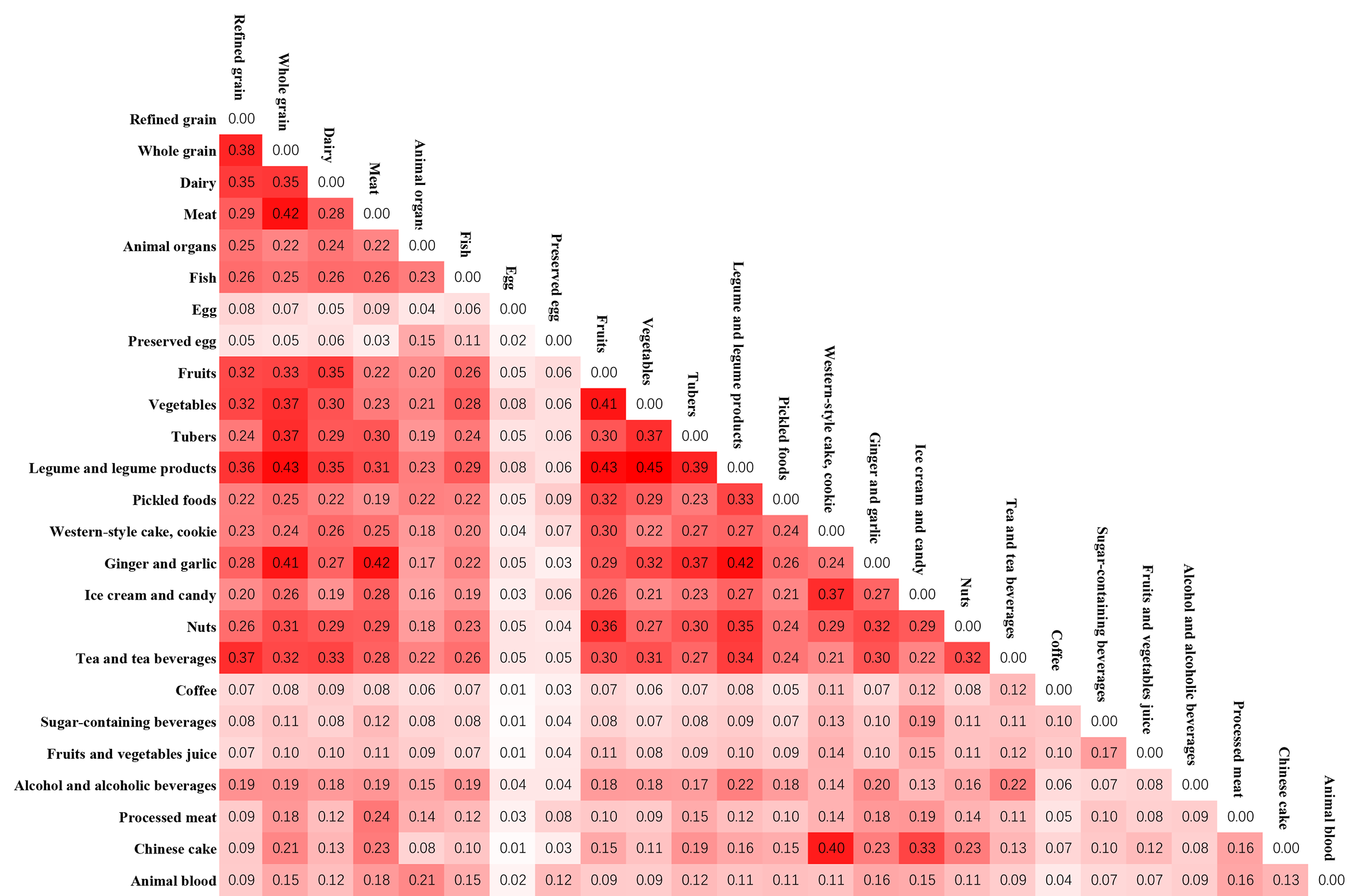

Supplement: Supplementary Figure 1 — Mutual information matrix of cases. Light to dark colors indicate lower to higher values. [file Image_1.TIF]

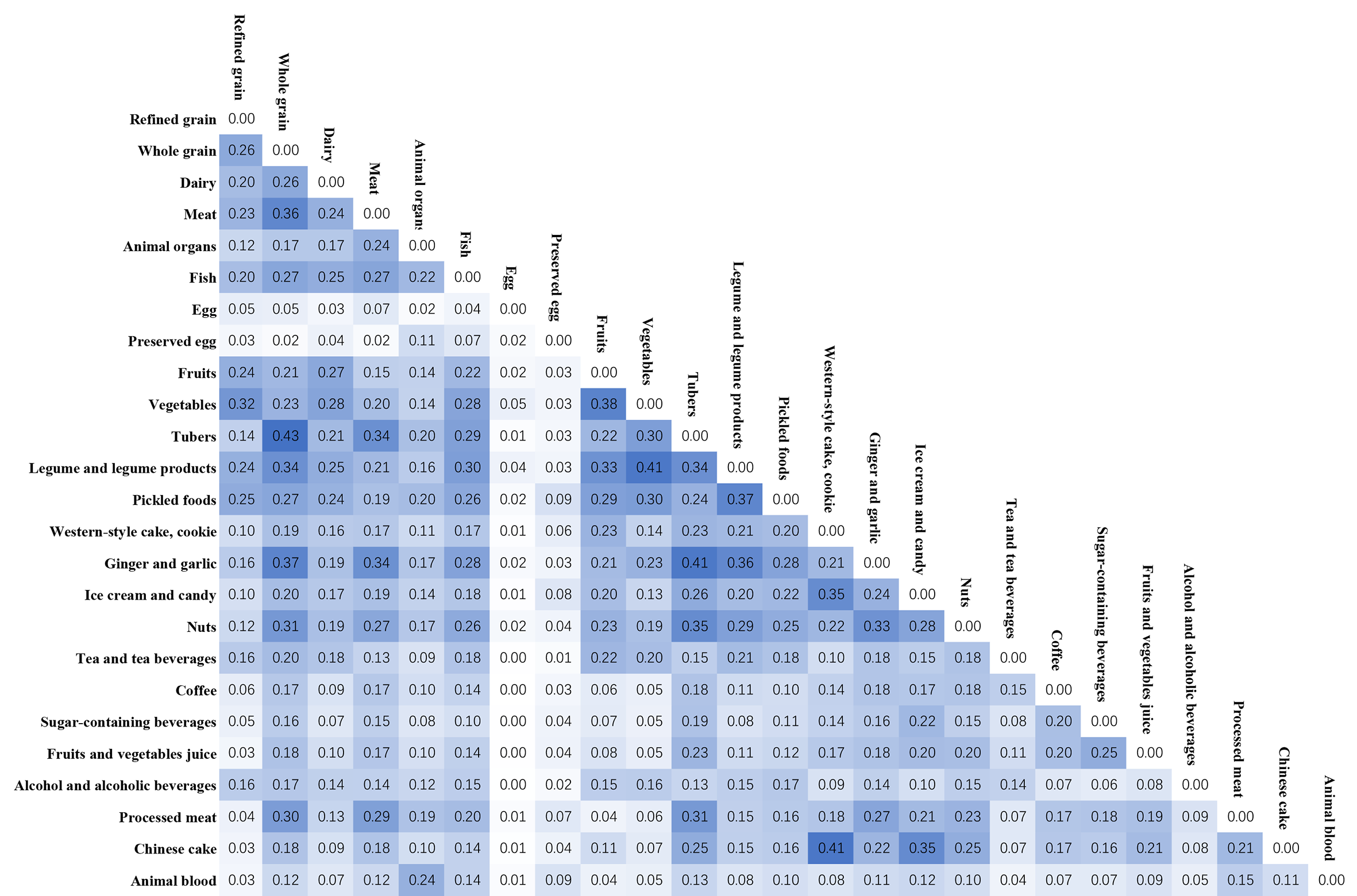

Supplement: Supplementary Figure 2 — Mutual information matrix of controls. Light to dark colors indicate lower to higher values. [file Image_2.TIF]
