# Supplementary material for: Complex Dietary Topologies in Non-alcoholic Fatty Liver Disease: A Network Science Analysis
Source: Front Nutr. 2020 Sep 29;7:579086. doi: 10.3389/fnut.2020.579086 (PMC7557363; doi:10.3389/fnut.2020.579086)
Supplement: Supplementary Table 1 — Participant characteristics by NAFLD status before matchinga. [file Table_1.DOCX]

Supplementary Table 1. Participant characteristics by NAFLD status before matching ^a^.

| Characteristics | NAFLD status | |  |
| --- | --- | --- | --- |
|  | No (n = 10460) | Yes (n = 3008) | *P* value ^b^ |
| Sex (male %) | 41.5 | 73.8 | <0.0001 |
| Age (y) | 40.8 (40.6, 41.0) ^c^ | 44.5 (44.1, 44.9) | <0.0001 |
| BMI | 22.9 (22.8, 23.0) | 27.8 (27.7, 27.9) | <0.0001 |
| Metabolic syndromes (%) | 11.6 | 58.1 | <0.0001 |
| Physical activity (Mets × hours/week) | 9.3 (9.1, 9.6) | 9.6 (9.1, 10.1) | 0.33 |
| Energy intake (kJ/d) | 8198.8 (8154.9, 8243.2) | 8310.2 (8227.3, 8393.9) | 0.02 |
| Education (≥college graduate, %) | 64.3 | 51.3 | <0.0001 |
| Household income (≥10,000 Yuan, %) | 35.1 | 34.8 | 0.79 |
| ALT (U/L) | 16.8 (16.5, 17.1) | 32.7 (32.1, 33.3) | <0.0001 |
| AST (U/L) | 18.0 (17.7, 18.4) | 22.4 (21.8, 23.0) | <0.0001 |
| GGT (U/L) | 23.3 (21.4, 25.2) | 49.4 (45.9, 52.9) | <0.0001 |
| Smoking status (%) |  |  |  |
| Smoker | 16.0 | 33.1 | <0.0001 |
| Ex-smoker | 3.7 | 7.3 | <0.0001 |
| Non-smoker | 80.4 | 59.6 | <0.0001 |
| Drinker (%) |  |  |  |
| Everyday | 4.3 | 9.2 | <0.0001 |
| Sometime | 53.6 | 60.9 | <0.0001 |
| Ex-drinker | 8.5 | 8.5 | 0.95 |
| Non-drinker | 33.6 | 21.5 | <0.0001 |
| Employment status (%) |  |  |  |
| Managers | 43.8 | 37.7 | <0.0001 |
| Professionals | 17.0 | 17.2 | 0.83 |
| Other | 39.2 | 45.1 | <0.0001 |
| Family history of diseases (%) |  |  |  |
| CVD | 29.8 | 27.0 | <0.01 |
| Hypertension | 48.5 | 50.8 | 0.03 |
| Diabetes | 22.6 | 26.2 | <0.0001 |

^a^ NAFLD, non-alcoholic fatty liver disease; CVD, cardiovascular disease; BMI, body mass index; ALT, alanine aminotransferase; AST, aspartate aminotransferase; GGT, γ-glutamyl transpeptidase.

^b^ Analysis of variance or chi-square test

^c^ Least square mean (95% confidence interval) (all such values)
