# Supplementary material for: Complex Dietary Topologies in Non-alcoholic Fatty Liver Disease: A Network Science Analysis
Source: Front Nutr. 2020 Sep 29;7:579086. doi: 10.3389/fnut.2020.579086 (PMC7557363; doi:10.3389/fnut.2020.579086)
Supplement: Supplementary Table 2 — Strengths and hubs for food groups according to NAFLD statusa. [file Table_2.DOCX]

Supplementary Table 2. Strengths and hubs for food groups according to NAFLD status ^a^.

|  | Strengths | | Hubs | |
| --- | --- | --- | --- | --- |
|  | Case | Control | Case | Control |
| Mean | 2.32 | 2.40 | 0.37 | 0.41 |
| Refined grain | 2.37 | 1.88 | 0.41 | 0.40 |
| Whole grain | 3.82 | 4.58 | 0.68 | 0.83 |
| Dairy | 1.77 | 1.87 | 0.30 | 0.38 |
| Meat | 2.57 | 2.89 | 0.45 | 0.50 |
| Animal organs | 2.55 | 1.81 | 0.32 | 0.21 |
| Fish | 3.40 | 2.61 | 0.58 | 0.45 |
| Egg | 0.35 | 0.36 | 0.06 | 0.07 |
| Preserved egg | 0.64 | 0.77 | 0.08 | 0.09 |
| Fruits | 2.28 | 2.91 | 0.37 | 0.51 |
| Vegetables | 3.48 | 4.28 | 0.55 | 0.78 |
| Tubers | 5.96 | 5.33 | 1.00 | 1.00 |
| Legume and legume products | 4.25 | 3.85 | 0.66 | 0.74 |
| Pickled foods | 2.35 | 2.89 | 0.40 | 0.51 |
| Western-style cake, cookie | 2.56 | 1.99 | 0.28 | 0.28 |
| Ginger and garlic | 4.05 | 2.80 | 0.74 | 0.62 |
| Ice cream and candy | 1.80 | 2.63 | 0.21 | 0.33 |
| Nuts | 1.66 | 2.76 | 0.36 | 0.55 |
| Tea and tea beverages | 1.38 | 1.53 | 0.26 | 0.24 |
| Coffee | 1.03 | 1.42 | 0.12 | 0.18 |
| Sugar-containing beverages | 1.41 | 1.76 | 0.16 | 0.19 |
| Fruits and vegetables juice | 1.68 | 1.45 | 0.17 | 0.20 |
| Alcohol and alcoholic beverages | 0.96 | 1.07 | 0.18 | 0.21 |
| Processed meat | 2.84 | 2.61 | 0.50 | 0.50 |
| Chinese cake | 1.75 | 2.75 | 0.23 | 0.35 |
| Animal blood | 0.97 | 1.17 | 0.15 | 0.14 |

^a^ NAFLD, non-alcoholic fatty liver disease
